# Microstructure, Hardness, and Elastic Modulus of a Multibeam-Sputtered Nanocrystalline Co-Cr-Fe-Ni Compositional Complex Alloy Film

**DOI:** 10.3390/ma14123357

**Published:** 2021-06-17

**Authors:** Péter Nagy, Nadia Rohbeck, Zoltán Hegedűs, Johann Michler, László Pethö, János L. Lábár, Jenő Gubicza

**Affiliations:** 1 Department of Materials Physics, Eötvös Loránd University, P.O. Box 32, H-1518 Budapest, Hungary; nagyp@student.elte.hu (P.N.); labar.janos@ek-cer.hu (J.L.L.); 2 EMPA Swiss Federal Laboratories for Materials Science and Technology, Laboratory for Mechanics of Materials and Nanostructures, Feuerwerkerstrasse 39, CH-3602 Thun, Switzerland; Nadia.Rohbeck@empa.ch (N.R.); johann.michler@empa.ch (J.M.); laszlo.petho@empa.ch (L.P.); 3 Deutsche Elektronen-Synchrotron DESY, Notkestr. 85, 22603 Hamburg, Germany; zoltan.hegedues@desy.de; 4 Institute for Technical Physics and Materials Science, Centre for Energy Research, Konkoly Thege Miklós út 29-33, H-1121 Budapest, Hungary

**Keywords:** multiple-beam-sputtering physical vapor deposition, compositional complex alloy, microstructure, hardness, elastic modulus

## Abstract

A nanocrystalline Co-Cr-Ni-Fe compositional complex alloy (CCA) film with a thickness of about 1 micron was produced by a multiple-beam-sputtering physical vapor deposition (PVD) technique. The main advantage of this novel method is that it does not require alloy targets, but rather uses commercially pure metal sources. Another benefit of the application of this technique is that it produces compositional gradient samples on a disk surface with a wide range of elemental concentrations, enabling combinatorial analysis of CCA films. In this study, the variation of the phase composition, the microstructure (crystallite size and defect density), and the mechanical performance (hardness and elastic modulus) as a function of the chemical composition was studied in a combinatorial Co-Cr-Ni-Fe thin film sample that was produced on a surface of a disk with a diameter of about 10 cm. The spatial variation of the crystallite size and the density of lattice defects (e.g., dislocations and twin faults) were investigated by X-ray diffraction line profile analysis performed on the patterns taken by synchrotron radiation. The hardness and the elastic modulus were measured by the nanoindentation technique. It was found that a single-phase face-centered cubic (fcc) structure was formed for a wide range of chemical compositions. The microstructure was nanocrystalline with a crystallite size of 10–27 nm and contained a high lattice defect density. The hardness and the elastic modulus values measured for very different compositions were in the ranges of 8.4–11.8 and 182–239 GPa, respectively.

## 1. Introduction

High-entropy alloys (HEAs) are in the focus of materials science. HEAs are equimolar or near-equimolar alloys formed from five or more elements that display high mixing entropy [1,2]. HEAs display many impressive attributes, such as outstanding strength and hardness, and excellent corrosion resistance [3,4,5,6,7]. In recent years, studies about HEA thin films started to appear more frequently in the literature. The manufacturing of HEA thin films utilizes many methods and techniques, such as direct-current magnetron sputtering [7,8], thermal evaporation [9], radio-frequency sputtering [10], or multibeam sputtering [11]. HEA thin films attract attention due to their immense potential in applications on account of their outstanding mechanical properties. In recent works, HEA thin films were compared with bulk materials with the same composition, the hardness and Young’s modulus measured for thin films often surpassed the values obtained on the bulk materials [11,12]. Many studies focused on the investigation of the microstructure of HEA thin films [8,13,14,15]. In the work of Dolique et al., it was shown that in the case of a magnetron-sputtered Al-Co-Cr-Cu-Fe-Ni HEA thin-film sample, even a small difference in the stoichiometry could lead to a different structure and thermal stability [13]. In the case of an Al-Co-Cr-Cu_0.5_-Fe-Ni thin film deposited by radio-frequency magnetron sputtering, it was shown how the sputtering condition could be used to tailor the microstructure and the chemical composition of the sample [10].

The advances in the field of HEAs inspired the investigation of other similar branches of materials. Compositionally complex alloys (CCAs) or multiprincipal element alloys (MPEAs) are a broader category of alloys that could contain fewer principal elements than a HEA material or differ in other ways. CCAs quickly became a thriving field of materials science due to the outstanding mechanical properties of these materials. The investigation of Al-Ti-V-Cr CCA revealed an exceptional corrosion resistance [16], and other studies reported remarkably high hardness values [4,17]. The effect of the chemical composition on the mechanical properties and the microstructure is one of the main foci of many works. Therefore, combinatorial samples provide an excellent opportunity to study many chemical compositions on one sample. In recent years, studies reported successful synthesis of combinatorial thin-film samples of CCAs [11,18,19].

In the present study, a combinatorial Co-Cr-Fe-Ni CCA thin film was manufactured by multiple-beam sputtering (MBS). MBS is a novel PVD method that does not require preliminary production of CCA targets, but instead uses commercially pure metal targets. Due to this feature, the samples synthesized via MBS exhibit well-defined element gradients, thus providing ideal samples to investigate the effect of chemical composition. The motivation of the study of the Co-Cr-Fe-Ni CCA system is that this can be regarded as the base alloy of many HEAs, such as the most-studied Cantor alloy (CoCrFeMnNi) [4,12,13,19]. Since the majority of CCAs based on Co-Cr-Fe-Ni have a face-centered cubic (fcc) structure, they therefore exhibit lower hardness than the body-centered cubic (bcc) CCAs. At the same time, the Co-Cr-Fe-Ni CCA family has been intensively studied in the literature, as they have good ductility and fracture toughness even at cryogenic temperatures. In addition, deviations from the equimolar composition may result in a change of the fcc crystal structure to other phases, such as bcc or hexagonal close-packed (hcp) structure, or even multiphase microstructures may form. The change of the phase composition and the microstructure due to tuning chemical composition can help to tailor the mechanical performance of materials in the Co-Cr-Fe-Ni CCA family. In this paper, the effect of the chemical composition on the microstructure and lattice-defect density, as well as the mechanical performance of a combinatorial Co-Cr-Fe-Ni CCA film, is investigated. The hardness and the elastic modulus of the layer are determined by nanoindentation, and their correlation to the phase composition and defect density is discussed.

## 2. Materials and Methods

### 2.1. Sample Preparation and Points of Interest

**Figure 1:**
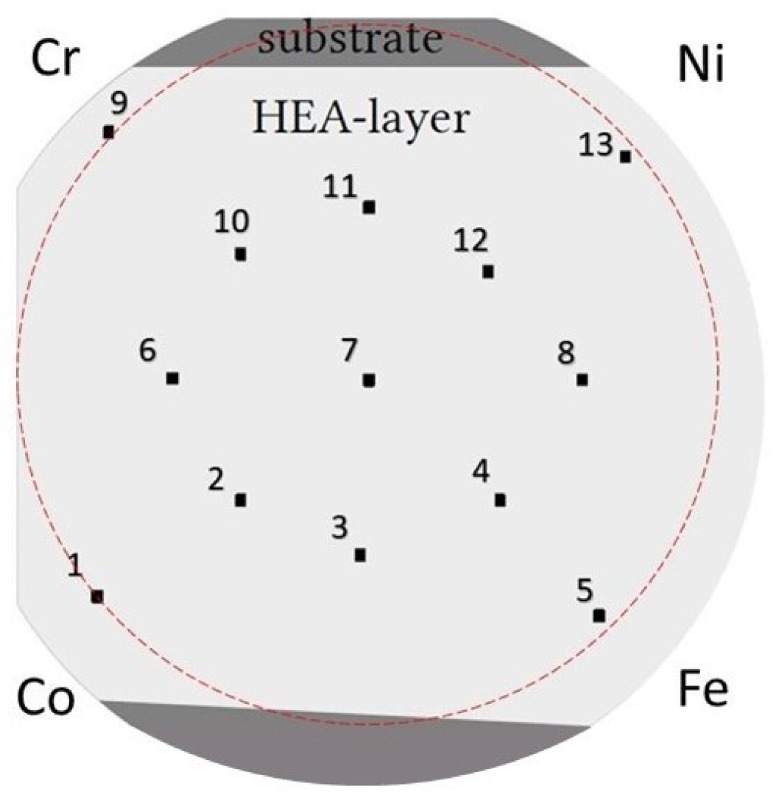
A schematic illustration of the studied locations (numbered from 1 to 13) on the Co-Cr-Fe-Ni thin film. The approximate positions of the four sputtering sources are also indicated. The dark grey areas show the parts of the Si substrate that were not covered by the film due to the masking effect of the sample holder. The dashed circle indicates the area for which concentration maps of the four constituent elements were prepared.

A recently introduced PVD technique was utilized for the preparation of the Co-Cr-Fe-Ni CCA thin-film sample. This technique is exceptional, since it uses 12 independent, commercially pure metallic targets for sputtering, three for each principal element (manufacturer: Polygon Physics, France). The targets were arranged around the substrate in a circle, and the same types of targets were placed next to each other. These attributes of the instrument led to a gradient of chemical composition in the sample. A more detailed description of this PVD method has been published elsewhere [11]. The substrate was a Si single crystal with a diameter of 10 cm. The as-deposited layer thickness was around 1 micron as measured by X-ray fluorescence (XRF) spectrometry (not shown here). The growing rate of the film thickness was very low (<100 nm/h), and the total deposition time was about 20 h. On the film surface, 13 points were selected for investigation, as shown in Figure 1. The point marked as No. 7 was close to the equimolar composition. Additional two–two points were selected equidistantly in the directions of the centers of the four PVD sources. These places were marked as 1, 2, 4, 5, 9, 10, 12, and 13. Moreover, four additional locations were investigated at the half radius of the disk between directions to the sources (marked as 3, 6, 8, and 11).

### 2.2. Analysis of the Chemical Composition

The chemical composition of the film at each point of interest was investigated by energy-dispersive X-ray spectroscopy (EDS) using a FEI Quanta 3D scanning electron microscope (SEM, manufacturer: Thermo Fisher Scientific, Waltham, MA, USA).

### 2.3. Investigation of the Microstructure

**Figure 2:**
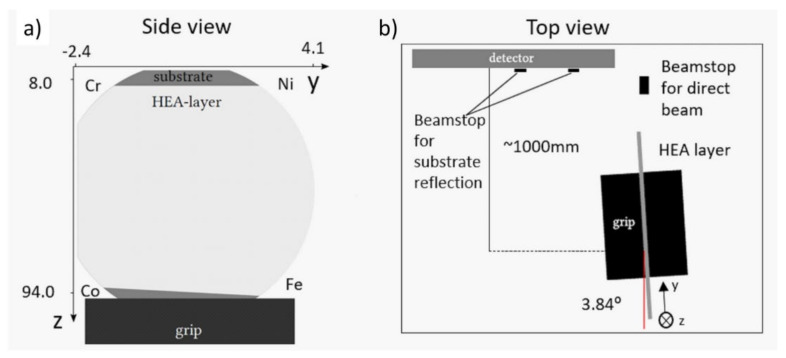
(**a**) Schematic side view of the sample disk showing the coordinate system used in the synchrotron XRD experiments for positioning of the beam on the locations of interest. (**b**) Schematic top view of the XRD setup.

The phase composition, the lattice constant, and the microstructure of the film at the points of interest were determined by synchrotron X-ray diffraction (XRD). The measurement was carried out at the Deutsches Elektronen-Synchrotron (DESY). The beam energy was set to 44 keV (λ = 0.028178 nm). The film had a strong 111 texture, as will be shown in Section 3. The inclination angle between the layer surface and the incoming beam was selected equal to the Bragg angle of reflection 111 (3.84°) shown in Figure 2. The beam size was set to 200 µm horizontally and 1000 µm vertically, resulting in a 1500 × 1000 µm^2^ spot size on the sample due to the low inclination angle of the incident beam. The diffraction rings were recorded with a Varex 4343 detector (pixel size: 150 µm, number of pixels: 2880 × 2880) placed 1 m behind the sample on the outboard side of the direct beam. The single-crystal reflections from the substrate were masked with round tungsten magnetic beamstops mounted directly in front of the detector. Due to the high energy of the beam and the very small probed volume at each studied point, six images were recorded at each position (the detector exposure time was limited to 5 s). Then, the intensities of the six patterns were summed up and integrated using the pyFAI software package (version 0.18, ESRF, Grenoble, France) [20] for obtaining diffractograms for evaluation.

The lattice constants were determined from the XRD peak positions using the Nelson–Riley method [21]. The microstructure in the 13 selected locations was characterized by X-ray line profile analysis (XLPA) [22]. The diffraction patterns were evaluated using convolutional multiple whole profile (CMWP) analysis [23]. In this method, the diffraction pattern is fitted by the convolution of the instrumental profile and the theoretical profiles derived from the crystallite size, dislocations, and twin faults (twin boundaries) summed with the background spline. The CMWP method yields the following parameters of the microstructure: the area-weighted mean crystallite size (<x>_area_), the dislocation density (ρ) and the twin fault probability (β). The latter quantity describes the fraction of the faulted {111} planes in fcc structures.

The multiphase microstructure of the film was also studied by transmission electron microscopy (TEM). A TEM-lamella was cut from the sample with a focused ion beam (FIB) technique using Ga ions. First, a trench around the lamella was dug using 30 keV and 30 nA until a lamella thickness of about 4 µm was obtained. Then, a thinning process was performed at a voltage of 30 keV and a current of 7 nA until a lamella thickness of 1.5 µm was achieved. Finally, the lamella was cut and transferred to a grid, where it was further thinned at 16 keV and 50 pA, followed by polishing at 5 keV and 48 pA, and finished at 2 keV and 27 pA. TEM bright-field (BF) and high-angle annular dark-field (HAADF) images were taken by a Titan Themis G2 200 transmission electron microscope (manufacturer: Thermo Fisher Scientific, Waltham, MA, USA). The TEM images were recorded at 200 keV with a 4k × 4k CETA 16 CMOS camera (Thermo Fisher Scientific, Waltham, MA, USA) controlled by VELOX software (version 2.14, Thermo Fisher Scientific, Waltham, MA, USA). A set of 50 × 50 diffraction patterns were recorded in microprobe STEM mode of the Themis, controlled by the TIA program. Automatic processing of these 2500 diffraction patterns and creation of the phase map from them was done using the ProcessDiffraction_V12.5.5 program, developed by one of the authors (J.L.L., to be published).

The crystallographic texture in the 13 locations was investigated by pole figure measurements using a Smartlab X-ray diffractometer (manufacturer: Rigaku, Tokyo, Japan) with CuKα radiation (wavelength: 0.15418 nm) with parallel-beam optics.

### 2.4. Nanoindentation

The mechanical performance of the film at the points of interest was investigated by nanoindentation using a Ubi nanoindenter equipped with a diamond Berkovich tip (manufacturer: Hysitron, Eden Prairie, MN, USA). The maximum load applied was 2 mN in order to guarantee a maximum penetration depth of ~70 nm, which was more than 10 times lower than the film thickness. The fulfillment of this condition was necessary for obtaining mechanical properties characteristic only to the film without the influence of the substrate. Both the loading and unloading parts of indentation took 5.6 s, and the holding time at the maximum load was 1.4 s. The hardness and the elastic modulus were determined by applying the Oliver–Pharr method [24]. Nine indentations were made in a 3 × 3 matrix at each location with a spacing of 10 µm.

## 3. Results

### 3.1. Chemical and Phase Composition in the Different Locations

**Figure 3:**
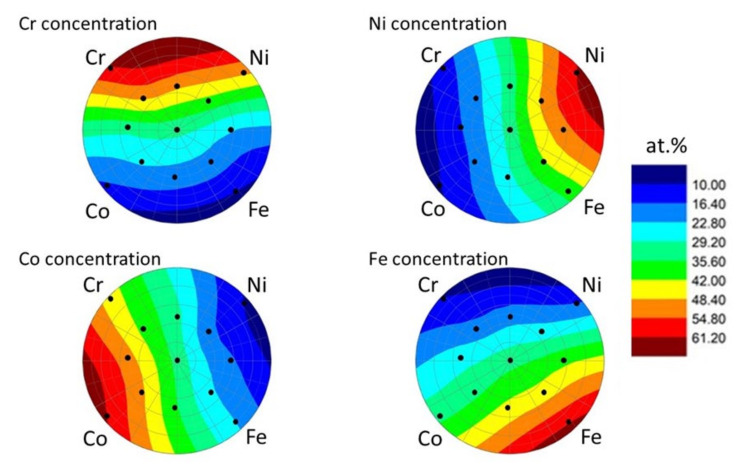
Concentration maps of the four constituent elements as obtained by SEM-EDS. The 13 studied locations are indicated by black spots.

The chemical compositions in the 13 locations of the Co-Cr-Ni-Fe film determined by SEM-EDS are listed in Table 1. The Cr, Fe and Ni contents varied between 5 and 44 at.% while the Co concentration was in the range of 12–62 at.%. The larger Co content can be explained by its higher sputtering rate. Each element was sputtered by three independent beams using separate targets grouped next to each other. The accumulated sputtering rates for Co, Cr, Fe, and Ni were measured to be 14.7, 10, 8.8, and 8.8 nm/hr, respectively. On the basis of the composition obtained in the 13 locations, concentration maps of the four constituent elements were prepared in the area indicated by the dashed circle in Figure 1. The edge of this circle was determined by the four outer locations among the 13 studied places. The concentration maps are shown in Figure 3, in which the atomic percentages between the studied points were estimated by interpolation using the contour plot with layer boundaries function in the software Origin. It should be noted that the mapping of the concentrations of the constituents was performed for another Co-Cr-Ni-Fe film in our previous paper [11]. That sample was processed under the same PVD conditions, and the chemical composition map was obtained on the whole disk by X-ray fluorescence (XRF) spectroscopy. In the present paper, the concentrations of the constituents were determined only in the 13 studied points using SEM-EDS. Figure 3 shows that the maximum concentrations for each element was shifted slightly clockwise away from the source, which can be explained by slight orientation uncertainties when the sample was placed into the PVD chamber. It should be noted that for each location, additional argon was detected with a concentration of 2–3 at.%. The Ar content did not show systematic variation as a function of the location in the layer. The source of argon was the sputtering beam. Due to the high energy of this beam (~10 keV), Ar atoms arrived at the substrate after the collision with the targets and deposited into the film. In addition, 1–2 at.% oxygen was also suggested by the EDS evaluation software for some studied locations; these values were very uncertain due to the strong overlapping of the peaks of oxygen and chromium in the EDS spectrum. Both argon and oxygen were excluded from the chemical composition given in Table 1.

**Figure 4:**
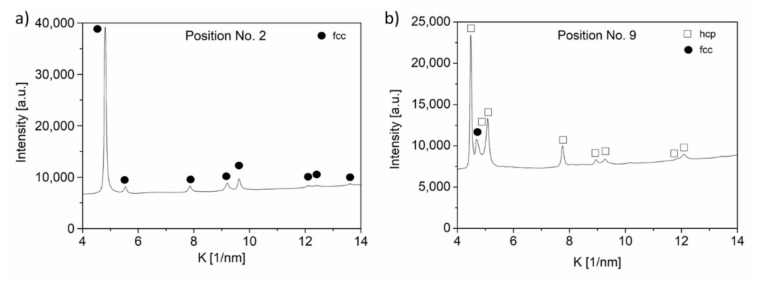
Diffraction patterns for point Nos. 2 (**a**) and 9 (**b**). The intensity is plotted as a function of the reciprocal space variable K = 2sinθ/λ, where θ is the Bragg angle and λ is the wavelength of X-rays.

The crystal structure determined by XRD is shown in Table 1. In almost all studied points, a single-phase fcc material was found. As an example, Figure 4a shows the diffraction pattern for the point No. 2, in which the intensity is plotted as a function of the reciprocal space variable K = 2sinθ/λ, where θ is the Bragg angle and λ is the wavelength of X-rays. At the same time, in location No. 9, with a composition of 43% Co–43% Cr–5% Fe–9% Ni (at.%), an hcp phase was identified as illustrated in Figure 4b. It should be noted that the second peak on this pattern seemed to be a sum of two close reflections, which most probably were reflection 002 of the hcp phase and peak 111 of a minor fcc phase. The dual phase composition at location No. 9 was also confirmed by TEM, as will be shown in the next section.

The lattice constants of the fcc and hcp phases were determined by XRD; they are listed in Table 1. As can be seen, the lattice constants of the fcc phases formed in the different locations were very similar; namely, they varied between 0.359 and 0.362 nm. Considering the experimental error of the lattice parameters (see Table 1), their values agreed within the experimental error despite the very different chemical compositions in the studied spaces. This observation can be explained by the very similar atomic radii of the constituent elements: 135 pm for Co and Ni, and 140 pm for Cr and Fe.

### 3.2. Microstructure as Determined by XLPA and TEM

**Figure 5:**
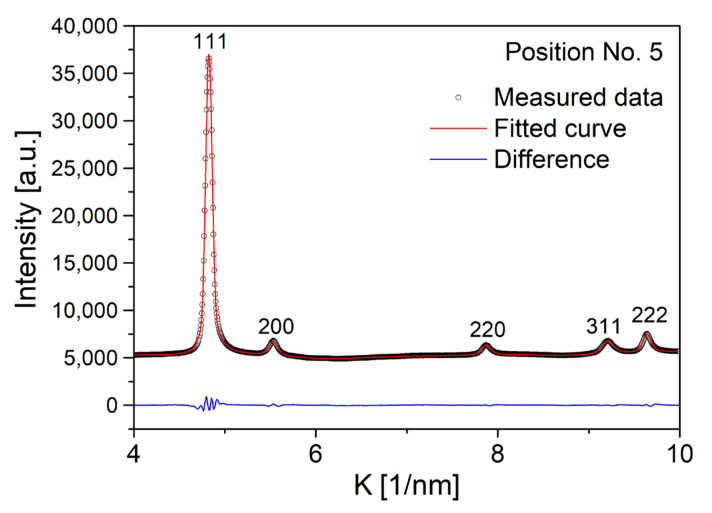
CMWP fitting for the first five XRD peaks of the fcc phase at point No. 5.

The microstructure was characterized by XLPA. As an example, Figure 5 shows CMWP fitting for the first five peaks of the fcc phase at point No. 5. The crystallite size, the dislocation density, and the twin-fault probability obtained from the fitting for the 13 locations are listed in Table 2. For point No. 9, the reflections of the hcp phase were evaluated by the CMWP method. In this case, the twin-fault probability was not determined, since for hcp structures, there are four different types of twins, and for the determination of the twin-fault probability, the type of twins must be known [22]. For the fcc phase, twin faults exist only on the {111} crystallographic planes.

**Figure 6:**
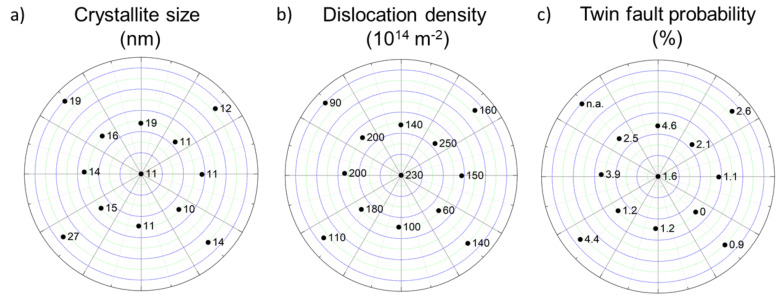
The crystallite size (**a**), the dislocation density (**b**), and the twin-fault probability (**c**) at different locations on the Co-Cr-Ni-Fe film.

Figure 6a–c show the crystallite size, the dislocation density, and the twin-fault probability at different locations in the Co-Cr-Ni-Fe film. It can be seen that the PVD-processed layer had a nanostructure at all studied points: the crystallite size varied between 10 and 27 nm. The dislocation density values of the different points were between 60 and 250 × 10^14^ m^−2^. Therefore, it can be concluded that a very high dislocation density with an order of magnitude of 10^16^ m^−2^ developed during the manufacturing of the CCA film. However, significant differences were found between the dislocation densities measured at different locations, so correlation between the chemical composition and the dislocation density could not be revealed. At the points with fcc structures, twin faults were also detected, except point No. 4. At other points, the twin-fault probability varied between 0.9 and 4.6%.

**Figure 7:**
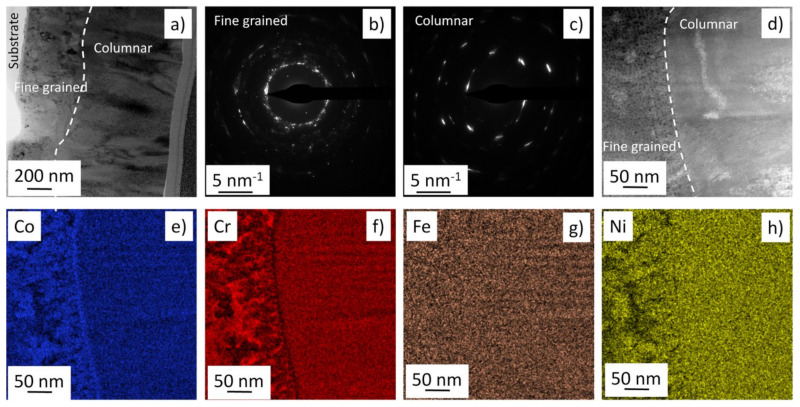
(**a**) Bright-field TEM image showing the cross-section of the layer in the vicinity of point No. 9 where the main phase was hcp. The white dashed curve indicates the boundary between the fine-grained and the columnar regions of the film. SAED patterns taken on the fine-grained (**b**) and columnar regions (**c**). (**d**) HAADF image of a magnified part of the film at the boundary between the fine-grained and columnar regions. The corresponding element maps for the four constituents are shown in (**e**–**h**).

In a previous paper [11], TEM investigations were performed on the cross-section of a PVD Co-Cr-Ni-Fe film at the place where the material exhibited equimolar composition and fcc structure. That layer was processed in the same way as the presently studied film, therefore in this study TEM investigation was carried out only on the place where the main phase was hcp structure (point No. 9). Our former TEM study on fcc structure revealed that the layer consisted of columns with a thickness of between 20 and 100 nm lying perpendicular to the surface of the film. It was also shown that the columns were fragmented into grains with a similar size as that of the crystallites determined by XLPA. The bright-field TEM image in Figure 7a shows the cross-section of the layer in the vicinity of point No. 9, where the main phase was hcp. The film thickness was about ~1.5 µm at this location. On the substrate side of the film, a fine-grained layer was found with a thickness of about 600 nm. The grain size varied between 20 and 70 nm in this part of the film. The other side of the layer contained coarser columnar grains with a thickness of about 100–200 nm (see Figure 7a). Figure 7b,c show selected area electron diffraction (SAED) patterns taken on the fine-grained and the columnar regions, respectively. For both locations, areas with diameters of about 500 nm were studied. The more continuous nature of the diffraction rings for the bottom part of the layer confirmed the finer grain size compared to the top part of the film. It should be noted that the grain sizes determined by the bright-field TEM images were somewhat larger than the crystallite size (~19 nm) obtained by XLPA. This observation suggests that the larger grains were fragmented into subgrains, since XLPA measures the coherently scattering domain size as crystallite size, and the coherency of X-rays breaks even for small misorientations. A magnified part of the film at the boundary between the fine-grained and columnar regions can be seen in the HAADF image in Figure 7d. The corresponding element maps for the four constituents are given in Figure 7e–h. Some chemical heterogeneities can be seen in the fine-grained region, while the columnar part of the film exhibited a fairly uniform distribution of the different elements.

**Figure 8:**
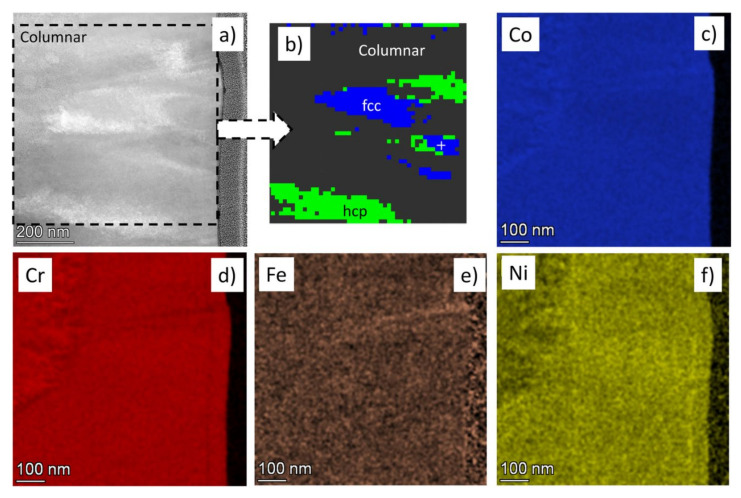
(**a**) HAADF image showing a magnified part of the layer in the columnar region at point No. 9. A phase map taken on this part of the sample can be seen in (**b**). The grey parts in (**b**) indicate areas where the phase identification was uncertain, while the green and blue regions are grains with hcp and fcc structures, respectively. The corresponding element maps for the four constituents are shown in (**c**–**f**).

The HAADF image in Figure 8a shows a magnified part of the layer in the columnar region at point No. 9. A phase map taken on this part of the sample can be seen in Figure 8b. The grey parts in Figure 8b indicate areas where the phase identification was uncertain, while the green and blue regions are grains with hcp and fcc structures, respectively. Most probably, the failure of the determination of phases in the grey areas was caused by the overlapping of the different phases in the thickness of the TEM lamella. The EDS element maps in Figure 8c–f revealed a high level of chemical homogeneity in the two phase regions; i.e., differences in the element concentrations between the hcp and fcc phases were not found at point No. 9.

**Figure 9:**
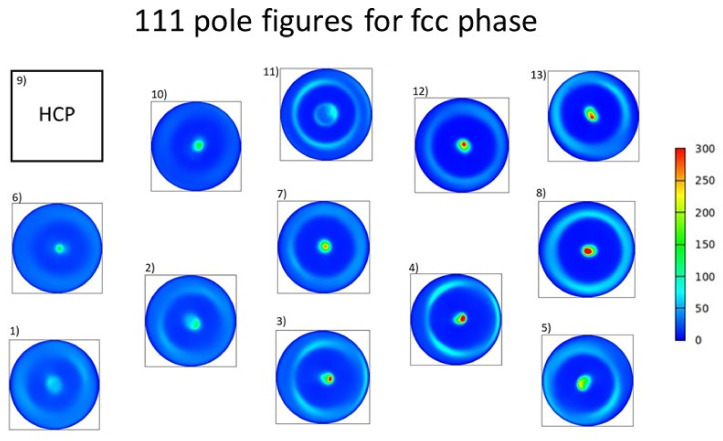
111 XRD pole figures characterizing the texture of the fcc phase in the different locations.

**Figure 10:**
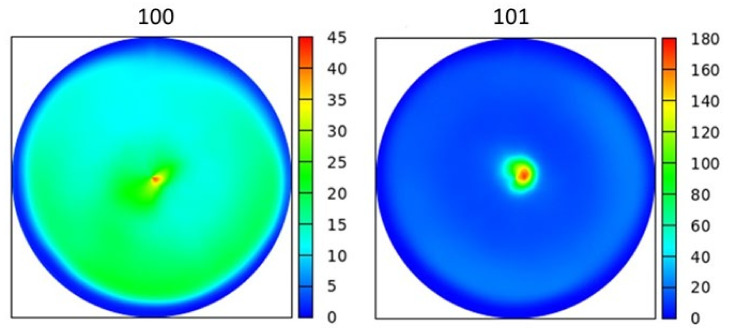
100 and 101 XRD pole figures characterizing the texture of the major hcp phase at point No. 9.

The crystallographic texture of the 12 locations having fcc structures was characterized by the 111 pole figures, as shown in Figure 9. For all points, 111 texture was observed; i.e., for most grains, the 111 lattice planes were parallel to the film surface. This crystallographic texture was in line with the very high intensity of the 111 peak in the XRD pattern shown in Figure 4a. At point No. 9, the texture of the major hcp phase was characterized by 100 and 101 pole figures (see Figure 10). The maximum intensity in the centers of the pole figures indicated that both 100 and 110 texture components existed in the material. This observation for the texture in the hcp phase was in accordance with the strong 100 and 101 reflection in the pattern shown in Figure 4b.

### 3.3. Mechanical Behavior from Nanoindentation

**Figure 11:**
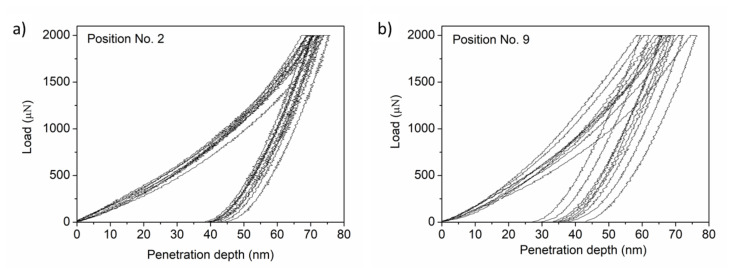
The load-depth curves obtained by nanoindentation for point Nos. 2 (**a**) and 9 (**b**).

**Figure 12:**
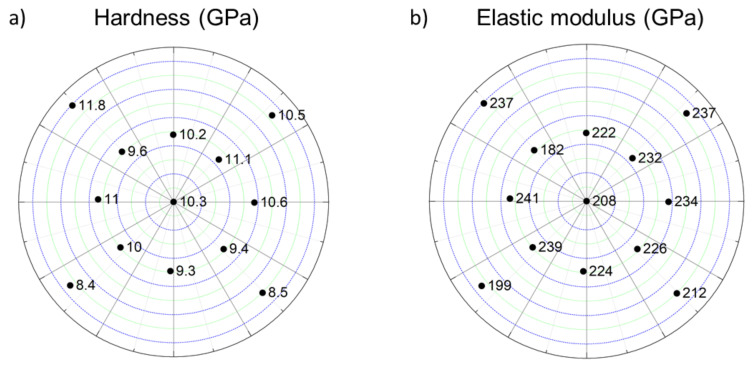
The hardness (**a**) and the elastic modulus (**b**) determined at different points on the Co-Cr-Ni-Fe film.

The mechanical performance of the PVD-processed Co-Cr-Ni-Fe film was investigated by nanoindentation. As an example, the load-depth curves for point Nos. 2 and 9 are shown in Figure 11a,b, respectively. For each location, nine indentations were made, therefore each figure contains nine curves. The hardness and the elastic modulus determined from the load-depth curves are listed in Table 3 and plotted in Figure 12. The hardness varied between 8.4 and 11.8 GPa, while the elastic modulus values were in the range of 182–241 GPa. However, there were significant differences between the mechanical parameters measured at different locations, so a correlation between the chemical composition, lattice defect densities, and mechanical behavior could not be easily established.

## 4. Discussion

The study of the 13 points on the Co-Cr-Ni-Fe thin film suggests that although the chemical composition varied in a wide range (between 5–7 and 41–44 at.% for Cr, Fe, and Ni, and between 12 and 62 at.% for Co), mainly an fcc structure formed. Indeed, for most of the probed points, only a single fcc phase was identified; the only exception was point No. 9, where the main phase was an hcp structure. Previous phase diagram calculations revealed that in the Co-Cr-Ni-Fe system, when all constituent element concentrations were between 20 and 40 at.%, a single-phase fcc structure formed at room temperature (RT) [25]. This condition was more or less fulfilled for point Nos. 7, 8, and 12 (see Table 1), therefore the formation of the fcc structure in these locations was reasonable.

Unfortunately, a full Co-Cr-Ni-Fe phase diagram describing the phases for compositions lower than 10 at.% at RT is not available in the literature. For instance, Gorsse and Senkov calculated the quaternary equilibrium phase diagram for the Co-Cr-Ni-Fe system only at 800 °C [26]. Since the present PVD processing was performed at RT, we tried to estimate the equilibrium phase composition from the room temperature ternary phase diagrams for the studied points where one or two components had relatively low concentrations (about 10 at.% or lower). For instance, for point Nos. 4 and 5, the compositions were close to CoFeNi and Co_2_Fe_4_Ni_3_, respectively. The Co-Fe-Ni ternary phase diagram [27,28] suggests a single fcc phase for these compositions, in accordance with the present experimental findings. Thus, in the area bounded by point Nos. 7, 4, 5, 8, and 12, the formation of an fcc phase was reasonable, according to the equilibrium phase diagrams. For other points, the phase diagrams did not predict a single fcc phase, but rather multiphase structures containing hcp, bcc, tetragonal, and orthorombic phases were suggested. For instance, in the case of the point No. 13, Cr and Ni were the two major elements with similar fractions. The Cr-Ni binary phase diagram revealed that for equimolar Cr-Ni alloy, the main phase is the orthorombic CrNi_2_ [29]. For location No. 1, the major elements were Co and Fe, with a concentration ratio of about 2:1. In this case, the Co-Fe phase diagram suggested that the material must contain a mixture of hcp and bcc phases [28]. At the same time, at this point, a single-phase fcc structure was observed. For location No. 9, the main constituents were Co and Cr at equal concentrations. The Co-Cr binary phase diagram predicted that below 100 °C, the solubility limits of Co in Cr and Cr in Co were practically zero, therefore nearly pure hcp Co and bcc Cr phases should coexist at RT in the equilibrium state of the material [30]. Slightly above 100 °C, a tetragonal Σ-phase is the major component of the microstructure for equimolar Co-Cr composition with a minor hcp component [31]. Thus, our experimental result suggesting hcp structure as the major phase was not in line with the prediction of the Co-Cr phase diagram. On the other hand, a recent study revealed that for a Co-Cr-Fe-Ni alloy in which Co and Cr were the major components (composition: Co_55_Cr_30_Fe_7.5_Ni_7.5_) after quenching from 1473 K to RT, most of the fcc phase (stable at high temperature) was transformed to hcp, and finally 75% of the alloy had an hcp structure [32]. Moreover, for CoCr alloys, the hcp phase is preferred at high pressures, even if additional alloying elements with high concentrations are added, such as in the case of the CoCrFeMnNi Cantor alloy [33]. Thus, it seems that under special processing conditions, the major phase may be hcp for Co- and Cr-rich compositions, similar to the present observation for point No. 9. For instance, the internal stresses in the PVD layer may yield the abundance of a Co- and Cr-rich hcp solid solution. Nevertheless, more research is needed for revealing the reasons of the development of the nonequilibrium phases in the present Co-Cr-Ni-Fe layer.

In this study, two mechanical parameters were determined for the 13 points on the Co-Cr-Ni-Fe thin film: the hardness and the elastic modulus. The present investigation revealed that both parameters changed significantly as a function of the position in the film: the largest variation could reach ~30% for both hardness and elastic modulus. This variation of the mechanical performance could be caused by the different chemical compositions and microstructures of the studied points. In addition, the change of the surface roughness could also yield variation in the mechanical performance, since our coating was not atomically flat. In addition, if there was a very small amount of porosity (well below 1%), the elastic modulus was reduced. The hardness was less susceptible to small-scale nanoporosity. At the same time, the hardness was significantly influenced by the density of dislocations, which had no considerable effect on the elastic modulus. The textures of the 12 fcc locations were very similar, therefore this effect on the variation of hardness and elastic modulus was negligible.

Due to the many effects influencing the hardness and the elastic modulus, a strict correlation between the chemical composition, the defect density, and the mechanical properties was not revealed in this study. However, it was evident that close to the Co and Fe sources, the hardness values were relatively low compared to the other parts of the PVD film (see Figure 12a). Namely, very close to the sources, the hardness was only 8.4–8.5 GPa, and even for point Nos. 1–5, the average hardness increased only to 9.1 GPa. At the same time, for locations between Nos. 6 and 13 (far from the Co and Fe sources) the average hardness was significantly higher: 10.6 GPa. The lower hardness close to the Co and Fe sources can be attributed mainly to the relatively low crystal-defect density (dislocations and twin faults) in the fcc phase.

It is worth noting that point No. 9, with an hcp structure as the main phase, had the highest hardness (~11.8 GPa) among the studied points. This observation cannot be attributed to the defect density or the crystallite size, since in this location the dislocation density was relatively low, while the crystallite size was among the largest compared to the other studied points (see Table 2 and Figure 6). Rather, the hcp crystal structure of the main phase could cause the high hardness, since the hexagonal structure is usually harder to deform by dislocation glide than the fcc materials, due to the low number of easy-slip systems in the former phase. It should also be noted that this hardness was exceptionally high among the values determined for Co-Cr-Ni-Fe materials in the literature. The hardness values published previously for the Co-Cr-Ni-Fe system are listed in Table 4. It can be seen, for instance, that the hardness of the magnetron-sputtered, nano-twinned CoCrFeNi thin film with equimolar constituent concentrations and fcc structure was about 8.5 GPa, which is similar to the lowest values obtained for the fcc locations in this study. At the same time, the location with the composition of 43% Co–43% Cr–5% Fe–9% Ni (at.%) and hcp structure exhibited a considerably higher hardness of ~11.8 GPa.

A future research direction will be to extend the mapping of the phase composition, defect density, and mechanical properties (hardness and elastic modulus) of the whole surface of the PVD-processed Co-Cr-Ni-Fe thin film. Most probably, close to the Fe source, a bcc structure also developed beside the regions with fcc and hcp phases. Nevertheless, this study suggests that although the crystal structures of the pure materials of the four element sources at ambient conditions were very different, the majority of the PVD film exhibited an fcc structure, even if the chemical composition varied in a wide range.

## 5. Conclusions

A combinatorial Co-Cr-Ni-Fe film was produced by multiple-beam-sputtering PVD on a silicon wafer. The chemical composition, the microstructure, and the mechanical properties of the layer were studied at 13 locations having very different constituent concentrations. The following conclusions were drawn from the results:

1. For the majority of the studied points on the layer, the material had a single-phase fcc structure with very similar lattice constants (0.359–0.362 nm), even if the concentrations of the constituents varied in a broad range. Namely, Cr, Fe, and Ni contents were between 5 and 44 at.%, while Co concentration varied in the range of 12–62 at.%. The larger Co content can be explained by its higher sputtering rate. For one location with a composition of 43% Co–43% Cr–5% Fe–9% Ni (at.%), the major phase was an hcp material with lattice constants of a = 0.258 nm and c = 0.417 nm. It can be concluded that the phase composition for many points in the CCA layer differed from the prediction of the available phase diagrams.
2. The crystallite size varied between 10 and 19 nm for most of the different compositions, and a larger value of 27 nm was found only for one location with the highest Co content (62 at.%). Therefore, we can state that a nanocrystalline microstructure developed in the sputtered Co-Cr-Ni-Fe film. The order of magnitude of the dislocation density was 10^16^ m^−2^ for all studied locations, although its value varied between 60 × 10^14^ m^−2^ and 250 × 10^14^ m^−2^, depending on the chemical composition. The twin-fault probability ranged between zero and 4.6% for the different studied points. A strict correlation between the chemical composition and the lattice-defect density was not found. For the fcc phase, a 111 crystallographic texture was developed in all studied locations. For the Co-Cr-rich point, the hcp phase had two texture components: 100 and 101.
3. A very high hardness with values between 8.4 and 11.8 GPa was measured for the different points of the Co-Cr-Ni-Fe layer, which could be caused by the nanocrystalline microstructure. It seemed that the variation of the crystal-defect (dislocations and twin faults) density had only a slight effect on the hardness. Rather, the chemical composition influenced the mechanical behavior, since the compositional dependence of the hardness was similar to that of the elastic modulus. The latter quantity varied between 182 and 239 GPa.

## Figures and Tables

**Table 1:** The coordinates of the studied positions (y and z), the chemical composition obtained by SEM-EDS, as well as the phase composition and the lattice constants determined by XRD.

No. of Position	y	z	Composition from SEM-EDS (at.%)				Phase Composition	Lattice Constant (nm)
			Co	Cr	Fe	Ni		
1	−1.5960	82	61	10	23	6	fcc	0.360 ± 0.001
2	−0.5240	70	52	14	23	11	fcc	0.360 ± 0.001
3	0.5480	76	40	11	32	17	fcc	0.360 ± 0.001
4	1.3520	70	26	12	32	30	fcc	0.360 ± 0.001
5	2.1560	84	21	7	41	31	fcc	0.359 ± 0.001
6	−1.0600	56	53	21	16	10	fcc	0.360 ± 0.001
7	0.5480	56	35	22	21	22	fcc	0.361 ± 0.001
8	2.1560	56	19	16	27	38	fcc	0.360 ± 0.001
9	−1.5960	26	43	43	5	9	hcp + (fcc)	a = 0.258 ± 0.001 c = 0.417 ± 0.001
10	−0.5240	42	43	32	12	13	fcc	0.362 ± 0.001
11	0.5480	36	28	34	13	25	fcc	0.362 ± 0.001
12	1.3520	44	22	28	16	34	fcc	0.361 ± 0.001
13	2.4240	30	12	33	11	44	fcc	0.361 ± 0.001

**Table 2:** The parameters of the microstructure (<x>_area_: area-weighted mean crystallite size; ρ: dislocation density; and β: twin-fault probability) determined by XLPA.

No. of Position	<x>_area_ (nm)	ρ (10^14^ m^−2^)	β (%)
1	27 ± 4	110 ± 10	4.4 ± 0.5
2	15 ± 2	180 ± 20	1.2 ± 0.1
3	11 ± 2	100 ± 10	1.2 ± 0.1
4	10 ± 2	60 ± 10	0 ± 0.1
5	14 ± 2	140 ± 20	0.9 ± 0.1
6	14 ± 2	200 ± 20	3.9 ± 0.4
7	11 ± 2	230 ± 30	1.6 ± 0.2
8	11 ± 2	150 ± 20	1.1 ± 0.1
9	19 ± 3	90 ± 10	n.a.
10	16 ± 2	200 ± 20	2.5 ± 0.3
11	19 ± 3	140 ± 20	4.6 ± 0.5
12	11 ± 2	250 ± 30	2.1 ± 0.2
13	12 ± 2	160 ± 20	2.6 ± 0.3

**Table 3:** The nanohardness (H) and the elastic modulus (E) determined by nanoindentation. The error was calculated as two times the standard deviation of 9 individual measurements.

No. of Position	H (GPa)	E (GPa)
1	8.4 ± 0.4	199 ± 8
2	10.0 ± 0.4	239 ± 5
3	9.3 ± 0.5	224 ± 5
4	9.4 ± 0.5	226 ± 6
5	8.5 ± 0.5	212 ± 7
6	11.0 ± 0.5	241 ± 6
7	10.3 ± 0.4	208 ± 5
8	10.6 ± 0.6	234 ± 6
9	11.8 ± 1.2	237 ± 12
10	9.6 ± 1.2	182 ± 14
11	10.2 ± 0.5	222 ± 7
12	11.1 ± 0.5	232 ± 6
13	10.5 ± 0.9	237 ± 10

**Table 4:** The hardness reported in the literature on materials in the Co-Cr-Ni-Fe system. SPS: spark plasma sintering; MA: mechanical alloying.

Composition	Processing Method	Grain Size	Load	Hardness (GPa)	Reference
Co16Cr51Fe17Ni15	atomized powder	<70 µm	2N	3.9	[34]
Co12Cr62Fe12Ni12	SPS	<70 µm	2N	2.3	[34]
Co14Cr52Fe15Ni15 + nickel-coated graphite and MoS2 powder	SPS	<70 µm	2N	3.7	[34]
CoCrFeNi	magnetron sputtering	7.8 nm	5 mN	8.5	[17]
Co27Cr22Fe24Ni127	SPS of gas atomized powder at 1000 °C	3–5 µm	3N	1.9	[35]
Co27Cr22Fe24Ni127	MA (500 rpm, 60 h) + SPS (1000 °C)	0.5–1 µm	3N	3.7	[35]
Co27Cr22Fe24Ni127	MA (500 rpm, 30 h) + SPS (1000 °C)	0.5–1 µm	3N	3.6	[35]
Co27Cr22Fe24Ni127	MA (500 rpm, 30 h) + SPS (950 °C)	0.5–1 µm	3N	3.5	[35]
Co27Cr22Fe24Ni127	MA (500 rpm, 30 h) + SPS (900 °C)	0.5–1 µm	3N	3.5	[35]
Co27Cr22Fe24Ni127	MA (250 rpm, 30 h) + SPS (1000 °C)	1–2 µm	3N	3.2	[35]
Co27Cr22Fe24Ni127	MA (250 rpm, 30 h) + SPS (950 °C)	1–2 µm	3N	3.4	[35]
Co27Cr22Fe24Ni127	MA (250 rpm, 30 h) + SPS (900 °C)	1–2 µm	3N	3.5	[35]
CoCrFeNi	arc melting	40 µm	n.a.	1.5	[36]
CoCrFeNi	arc melting (undercooling: 50 K)	30 µm	n.a.	1.9	[36]
CoCrFeNi	arc melting (undercooling: 1000 K)	15 µm	n.a.	2.1	[36]
CoCrFeNi	arc melting (undercooling: 150 K)	15 µm	n.a.	2.5	[36]
CoCrFeNi	arc melting (undercooling: 200 K)	15 µm	n.a.	2.5	[36]
CoCrFeNi	arc melting (undercooling: 250 K)	12 µm	n.a.	2.5	[36]
CoCrFeNi	arc melting (undercooling: 300 K)	7 µm	n.a.	2.7	[36]
CoCrFeNi	arc melting	250 µm	100 mN	3.6	[37]

## Data Availability

The evaluated data presented in this study are available in the tables of this paper. The raw measured data of this study are available on request from the corresponding author.
